# Impact of extended reality (XR) simulation on ophthalmology training outcomes: an updated systematic review and meta-analysis

**DOI:** 10.3389/fmed.2026.1823359

**Published:** 2026-05-01

**Authors:** Yuxi Jiang, Shengfang Ge, Bilian Ke

**Affiliations:** Department of Ophthalmology, Ren Ji Hospital, Shanghai Jiao Tong University School of Medicine, Shanghai, China

**Keywords:** augmented reality, cataract surgery, extended reality, meta-analysis, ophthalmic surgical training, systematic review, virtual reality

## Abstract

**Background:**

Extended reality (XR) simulation offers repeatable, risk-free practice for ophthalmic training. We conducted a systematic review and meta-analysis to quantify its effects on training outcomes.

**Methods:**

We searched MEDLINE (PubMed), Embase, Web of Science Core Collection, and Cochrane CENTRAL from inception to February 6, 2026. Risk of bias was assessed using RoB 2 (randomized trials) and ROBINS-I (non-randomized studies), and certainty of evidence was evaluated with GRADE. Statistical analysis was performed using Stata 15.

**Results:**

Thirty-two studies (1,572 trainees) were included. XR training significantly reduced overall intraoperative complications (OR = 0.72; 95% CI, 0.63–0.82; *P* < 0.001), posterior capsule rupture (OR = 0.63; 95% CI, 0.49–0.81; *P* < 0.001), and CCC-related complications (OR = 0.44; 95% CI, 0.21–0.90; *P* = 0.024). Vitreous loss and retained lens material did not differ significantly. Technical performance improved, with higher global surgical scores (SMD = 1.93; 95% CI, 1.49–2.38; *P* < 0.001) and CCC scores (SMD = 0.73; *P* < 0.001). Total operative time was significantly shortened (WMD = −8.92 min; 95% CI, −16.38 to −1.46; *P* = 0.019). Trainee confidence generally improved, though perceived realism varied.

**Conclusions:**

Extended reality (XR) simulation training can enhance ophthalmic surgical technical performance and procedural efficiency, reduce the risk of intraoperative complications, and improve trainees' operative confidence. These findings provide an evidence-based foundation for the design and optimization of ophthalmology training curricula. Future studies should further investigate the value of XR technologies in non-cataract procedures and in the development of non-technical skills.

**Systematic trial registration:**

https://www.crd.york.ac.uk/prospero/, identifier: CRD420261288204.

## Introduction

Global population aging is driving a steady increase in ophthalmic clinic visits and surgical volume (1, 2). Ophthalmology trainees therefore need to master diverse operative skills within limited training time to meet rising clinical demands (3, 4). Cataract surgery and other intraocular procedures demand high precision. Intraoperative errors, such as posterior capsule rupture with vitreous loss, increase the risk of cystoid macular edema, endophthalmitis, and retinal detachment, and may lead to permanent vision loss (5). As expectations for patient safety rise, novices have fewer opportunities to learn in live cases. At the same time, pressure to maintain operating-room efficiency and turnover reduces tolerance for prolonged cases for teaching, further limiting residents' opportunities to act as primary surgeons (6, 7). Apprenticeship-based instruction and wet-lab training remain essential, but it is increasingly difficult to ensure patient safety and surgical quality while also providing sufficient, structured practice (8, 9).

Since the 1990s, extended reality (XR), including virtual reality (VR) and augmented reality (AR), has been introduced into ophthalmic training to provide repeatable, measurable practice without adding risk to patients (10, 11). Microsurgical simulators such as the Eyesi system are used in training centers worldwide, and some experts recommend integrating them into standardized residency curricula (3–5). A national Delphi study identified 32 core modules for the Eyesi slit-lamp simulator, supporting curriculum-based implementation (4). High-fidelity platforms such as HelpMeSee focus on manual small-incision cataract surgery (MSICS) and offer centralized courses to shorten the learning curve for beginners (12). However, survey data indicate that use of XR simulation remains uneven: many programs do not require it, and trainee exposure varies widely. Acquisition and maintenance costs remain major barriers, especially in low- and middle-income countries and smaller programs. Even in equipped centers, scheduling constraints, limited faculty time, and institutional policies can restrict training volume, and approaches to implementation and assessment are not yet standardized (13–15). Therefore, the size of the benefit, the settings in which XR training is most effective, and the certainty of the evidence should be clarified through systematic synthesis.

Randomized controlled trials and prospective studies suggest that XR simulation improves technical performance, with higher scores and fewer errors on simulators and, in some settings, during live surgery (16, 17). Several studies also report lower rates of major cataract complications, including posterior capsule rupture and vitreous loss, after simulation-based training (18, 19). Existing systematic reviews and meta-analyses generally support improved technical performance and suggest potential gains in patient safety (20, 21). However, prior syntheses have focused mainly on cataract training or specific platforms, and evidence for other ophthalmic procedures and non-surgical skills remains limited. For several key outcomes, meta-analysis is still lacking, leaving uncertainty about the strength and applicability of the evidence. Given the rapid growth of the literature and increasing training needs, an updated and broader evaluation is warranted (22). In this study, we systematically synthesize interventional evidence on XR simulation in ophthalmology training to estimate its effects and to inform curriculum design and future development.

## Materials and methods

### Study design and registration

This study was conducted as a systematic review and meta-analysis. The study protocol was developed a priori before study initiation and was registered on the PROSPERO platform (registration number: CRD420261288204). Reporting of this review followed the PRISMA 2020 statement.

### Information sources and search strategy

We systematically searched the following databases from inception to February 6, 2026: MEDLINE (via PubMed), Embase, Web of Science Core Collection, and the Cochrane Central Register of Controlled Trials (CENTRAL). The search strategy was developed using a combination of controlled vocabulary terms (e.g., MeSH/Emtree) and free-text keywords, combined with Boolean operators (OR/AND). Search syntaxes were adapted to the specific requirements of each database, including its controlled vocabulary system and query structure.

Search terms included but were not limited to, concepts related to ophthalmic surgery/skills training and extended reality, such as “Ophthalmologic Surgical Procedures,” virtual reality, augmented reality, mixed reality, simulation, simulator, patient simulation, medical education, and Eyesi. To minimize the risk of missing eligible studies, we also manually screened the reference lists of included studies and relevant reviews. All search processes (databases, search dates, complete strategies, and numbers of records retrieved) were documented to ensure reproducibility. Full search results and strategies are provided in Supplementary Table 1 and Appendix 1.

### Inclusion and exclusion criteria

Inclusion criteria (PICO):

We included original studies evaluating the effects of extended reality simulation training on skill performance among ophthalmology trainees. Eligible studies were required to meet all of the following criteria:

Population: Ophthalmology-related trainees (e.g., medical students, residents, fellows, or visiting trainees).

Intervention: XR-based simulation training for ophthalmic surgery or ophthalmic clinical skills.

Comparator: Conventional training, no XR exposure, or a comparator group with lower XR training exposure.

Outcomes: Reporting at least one training-related outcome, such as Surgical Competence Scores, Intraoperative Complications, or learner-related outcomes (e.g., self-efficacy).

Additionally, all studies were original English articles.

Exclusion criteria:

Studies were excluded if they met any of the following conditions: (1) case reports, reviews, meta-analyses, letters to the editor, or commentaries; (2) conference abstracts without subsequent full-text publication (conference abstracts were included if a full-text article was later published and met the inclusion criteria); (3) Inability to obtain the full text; (4) animal studies.

### Study selection process

Two reviewers independently screened titles and abstracts identified through the searches. Records deemed potentially eligible proceeded to full-text review. Subsequently, the same two reviewers independently assessed full texts and made final inclusion decisions. Disagreements were resolved through discussion; if consensus could not be reached, a third reviewer adjudicated the disagreement. Multiple reports originating from the same study were identified and grouped accordingly.

### Data extraction

A standardized data extraction form was used and pilot-tested prior to formal extraction. Two reviewers independently extracted outcome data and cross-checked for accuracy, and key study characteristics were extracted or verified in duplicate whenever feasible. Extracted information included: (1) general information (authors, year of publication, country); (2) study design and sample characteristics (study type, sample size); (3) trainee characteristics (training stage, baseline experience); (4) intervention details (type of XR, simulator/platform name, training modules); (5) training content in the control group; (6) outcome definitions, measurement tools, and data (means/standard deviations or convertible metrics; event counts for dichotomous outcomes).

### Risk of bias assessment and certainty of evidence

The overall risk of bias for each included study was represented by the risk-of-bias judgment for its primary outcome. For randomized controlled trials (RCTs), we used the Cochrane Risk of Bias tool (RoB 2) and judged risk across five domains as “low risk,” “some concerns,” or “high risk.” For non-randomized intervention studies, we used ROBINS-I, which covers seven domains, and rated risk as “low,” “moderate,” “serious,” or “critical,” with the overall judgment determined by domain-level assessments. The certainty of evidence for each key outcome was evaluated using the GRADE (Grading of Recommendations, Assessment, Development and Evaluations) framework. Two reviewers independently conducted risk of bias and certainty assessments; disagreements were resolved by discussion and, when necessary, adjudicated by a third reviewer. Risk of bias assessments and the GRADE evidence profile are presented in Supplementary Figures 1–3.

### Statistical analysis and data synthesis

All analyses were performed using Stata 15.0. Continuous outcomes were summarized as weighted mean differences (WMDs) or standardized mean differences (SMDs), with 95% confidence intervals (CIs). Dichotomous outcomes were summarized as odds ratios (ORs) with 95% CIs. Statistical heterogeneity was assessed using Higgins' *I*^2^ statistic. Substantial heterogeneity was defined as *I*^2^ > 50%, in which case a random-effects model was applied; otherwise, a fixed-effect model was used. When substantial heterogeneity was present, sensitivity analyses and subgroup analyses were conducted to explore potential sources. Publication bias was visually assessed via funnel plots (≥10 studies included) and statistically evaluated using Egger's regression test (≥5 studies included). If publication bias was detected, the trim-and-fill method was applied to examine the potential impact of publication bias on pooled estimates. Statistical significance was set at a two-sided *P* < 0.05.

## Results

### Results of the search

**Figure 1:**
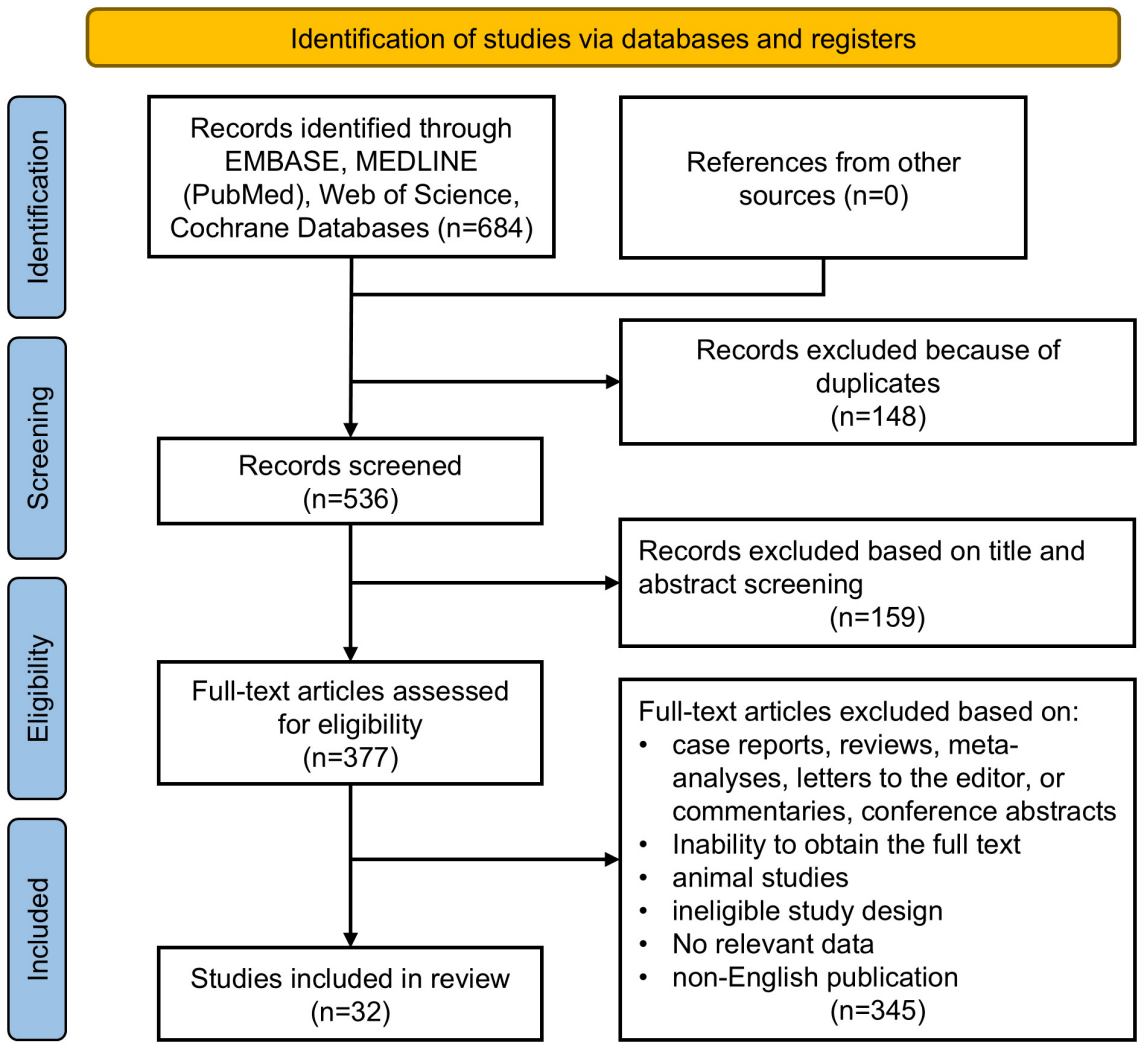
PRISMA flow diagram of study selection for the meta-analysis.

The systematic search and screening process is shown in Figure 1 (PRISMA flow diagram). A total of 684 records were identified. After removing duplicates, 536 records remained. Title and abstract screening retained 377 articles for full-text assessment. After full-text review, studies were excluded due to an ineligible study design, irrelevance to the topic, non-English publication, animal experiments, or insufficient usable data. Finally, 32 eligible studies were included, involving 1,572 participants. Of these, 14 studies were included in the quantitative synthesis, whereas the remaining studies were summarized narratively because meta-analysis was not feasible. The included studies were published between 1998 and 2026, and 31 studies (96.9%) were published after 2009.

### Study characteristics

Among the 32 included studies, there were 10 randomized controlled trials (RCTs, 31.3%), 10 retrospective cohort studies (31.3%), 8 single-arm studies (25.0%), and 4 non-randomized controlled studies (12.5%). The studies were conducted across multiple regions worldwide, most commonly in Europe (*n* = 13, 40.6%), followed by North America (*n* = 8, 25.0%) and Asia (*n* = 8, 25.0%). A smaller number of studies were conducted in Africa (*n* = 2, 6.3%) and South America (*n* = 1, 3.1%).

Regarding participants, 21 studies (65.6%) primarily enrolled ophthalmology residents, spanning training levels from PGY-1 to PGY-5. Six studies (18.8%) targeted medical students, and the remaining five studies (15.6%) involved practicing ophthalmologists, fellows, or mixed-experience cohorts.

With respect to XR devices, the Eyesi Surgical simulator was the most widely used platform, reported in 25 studies (78.1%). Other devices included the HelpMeSee simulator (*n* = 3, 9.4%), and single studies using the PixEye simulator (3.1%), SOPHOCLE simulator (3.1%), MicroREC AR system (3.1%), and a screen-based desktop VR system (3.1%).

In terms of training content, cataract surgery was the main focus. Specifically, 24 studies (75.0%) evaluated phacoemulsification (Phaco) and key steps such as continuous curvilinear capsulorhexis (CCC) and nucleus cracking/chopping. Three studies focused on manual small-incision cataract surgery (MSICS). In addition, one study each investigated laser trabeculoplasty, panretinal photocoagulation, pars plana vitrectomy, and slit-lamp examination, and one study specifically targeted basic microsurgical skills. Overall, most studies reported that simulator-based training was associated with higher skill scores, shorter procedure time, or lower complication rates. Detailed study characteristics, device specifications, and outcomes are summarized in Table 1 and Supplementary Table 2.

**Table 1:** Study characteristics of included studies.

Study ID	Country	*N*	Population	Study design	Simulation modality	Intervention (Simulation group)	Control (Comparison group)	Assessment platform	Target procedure
Adnane, 2020 (33)	Morocco	12	Residents (Year 3)	RCT	VR (Eyesi surgical)	Completed a total of 30 h of Eyesi simulator phacoemulsification training curriculum before the resident's first live surgery.	Received only traditional residency training without simulator exposure, proceeding directly to live surgery.	Live surgery	Cataract Phaco (Full procedure)
Alwadani, 2012 (23)	Saudi Arabia	47	Medical students (Year 5)	Non-RCT	VR (PixEye simulator)	Received PixEye simulator training until proficiency criteria were met, combined with theoretical PowerPoint lectures.	Received only the same theoretical PowerPoint lectures, without any simulator practice.	VR simulator (PixEye)	Laser trabeculoplasty
Belyea, 2011 (30)	USA	42	Residents (PGY-3)	Retrospective cohort	VR (Eyesi surgical)	Access to Eyesi simulator practice during residency training.	Retrospective analysis of resident surgical data prior to the introduction of the simulator.	Live surgery	Cataract Phaco (Full procedure)
Bergqvist, 2014 (17)	Denmark	20	Medical Students (Novice)	RCT	VR (Eyesi surgical)	Completed the full Eyesi cataract training curriculum once weekly for 4 consecutive weeks.	Completed training only in Week 1 and Week 4, with no practice in the intervening 2 weeks (Low-dose control).	VR simulator (Eyesi)	Cataract modules (Capsulorhexis, Phaco, Navigation)
Daly, 2013 (34)	USA	16	Residents (PGY-2)	RCT	VR (Eyesi surgical)	Repeated practice of capsulorhexis (CCC) on the Eyesi simulator preoperatively until passing the specified curriculum standards.	Repeated capsulorhexis practice on synthetic eyes (Kitaro Dry-lab) preoperatively until subjectively confident.	Live surgery	Cataract Phaco (Continuous Curvilinear Capsulorhexis CCC)
Deuchler, 2016 (28)	Germany	20	Vitreoretinal surgeons	RCT	VR (Eyesi surgical)	Performed approximately 20 min of targeted warm-up training on Eyesi immediately before performing vitrectomy.	No preoperative simulator warm-up, proceeding directly to surgery.	Live surgery	Pars Plana vitrectomy
Deuchler, 2023 (29)	Germany	24	Medical students (Year 4)	RCT	VR (Eyesi slit lamp)	Received Eyesi slit lamp simulator training during a 1-week ophthalmology clerkship.	Participated only in the 1-week traditional ophthalmology clerkship (including peer-to-peer practice), without simulator.	Clinical skills exam	Slit lamp examination
Ducloyer, 2024 (26)	France	16	Residents (PGY-1/Novice)	Single-arm Pre-post	VR (Eyesi surgical)	Completed one 2-h Eyesi simulator training session daily for 4 consecutive days (total 8 h).	N/A (Single-arm observational learning curve study).	VR simulator (Eyesi)	Cataract Phaco (Full procedure)
Eltanamly, 2022 (36)	Egypt	30	Surgeons (Intermediate/Expert)	Single-arm (Self-control)	VR (Eyesi surgical)	Completed 3 consecutive capsulorhexis tasks on Eyesi using the non-dominant hand.	Completed the same 3 tasks using the dominant hand (within-subject control).	VR simulator (Eyesi)	Cataract Phaco (Continuous Curvilinear Capsulorhexis CCC)
Ferris, 2020 (43)	UK	265	Trainees (ST1 & ST2)	Retrospective cohort	VR (Eyesi surgical)	Trainees at centers possessing and using Eyesi simulators for teaching.	Trainees at centers without Eyesi simulators (based on national database comparison).	Live surgery	Cataract Phaco (Full procedure)
Feudner, 2009 (44)	Germany	63	Students & Residents	RCT	VR (Eyesi surgical)	Received 2 rounds of Eyesi simulator intensive training between pre- and post- wet-lab assessments.	No additional practice between pre- and post- wet-lab assessments.	Wet-lab (Porcine Eye)	Cataract Phaco (Continuous Curvilinear Capsulorhexis CCC)
Gonzalez, 2016 (31)	USA	14	Attending & Trainees	Single-arm pre-post	VR (Eyesi surgical)	Completed a full curriculum comprising baseline testing, 3 repeated training blocks, and terminal testing.	N/A (Comparison of pre-test vs. post-test scores).	VR simulator (Eyesi)	Cataract Phaco (Continuous Curvilinear Capsulorhexis CCC)
Hu, 2021 (27)	China	60	Residents (Year 2)	RCT	VR (Eyesi surgical)	Repeated nuclear chopping practice 10 times on the Eyesi simulator.	Repeated nuclear chopping practice 10 times on porcine eyes in a wet-lab.	Dual (VR & Wet-lab)	Cataract Phaco (Chopping)
Le, 2024 (24)	Denmark	29	Ophthalmologists (No MSICS exp)	Non-RCT	VR (HelpMeSee)	Surgeons who had previously passed the Eyesi phacoemulsification curriculum proficiency standards.	Surgeons with no prior Eyesi training experience and no phacoemulsification surgical experience.	VR Simulator (HelpMeSee)	MSICS (Manual Small Incision Cataract Surgery)
Lopez, 2020 (45)	USA	29	Residents (PGY-4)	Retrospective cohort	VR (Eyesi surgical)	Mandatory completion of the specified Eyesi training curriculum during the second year of residency (PGY-2).	Retrospective analysis of resident data prior to mandatory curriculum implementation (no simulator training).	Live surgery	Cataract Phaco (Full procedure)
Lucas, 2019 (46)	Brazil	14	Residents (Year 2)	Retrospective cohort	VR (Eyesi surgical)	Completed the Eyesi simulator training curriculum.	Retrospective analysis of resident data prior to simulator introduction.	Live surgery	Cataract Phaco (Full procedure)
Mathis, 2022 (32)	France	24	Residents (Year 1)	Single-arm Pre-post	VR (Eyesi surgical)	Mandatory “Cataract Challenge” mode (including high-tension capsulorhexis) performed after every 1 h of basic training.	N/A (Assessment of learning curve and time to proficiency).	VR simulator (Eyesi)	Cataract Phaco (Cataract Challenge Mode)
McCannel, 2013 (47)	USA	38	Residents (PGY-3 & 4)	Retrospective cohort	VR (Eyesi surgical)	Completed the “Capsulorhexis Intensive Training Curriculum” (CITC) until reaching a specific score threshold.	Historical control: residents trained prior to the implementation of the CITC curriculum.	Live surgery	Cataract Phaco (Continuous Curvilinear Capsulorhexis CCC)
McCannel, 2017 (48)	USA	38	Residents (PGY-3 & 4)	Retrospective cohort	VR (Eyesi surgical)	Completed the “Capsulorhexis Intensive Training Curriculum” (CITC) until reaching a specific score threshold.	Historical control: residents trained prior to the implementation of the CITC curriculum.	Live surgery	Cataract Phaco (Full procedure/Vitreous loss)
Montrisuk., 2022 (19)	Thailand	41	Residents (Year 3)	Retrospective cohort	VR (Eyesi surgical)	Completed a mandatory surgical simulation training course.	Group of residents who did not complete the simulation course.	Live surgery	Cataract Phaco (Full procedure)
Muth, 2026 (49)	Germany	21	Trainees & Experts	Non-RCT	AR (MicroREC System)	Porcine eye capsulorhexis training assisted by the MicroREC Augmented Reality (AR) overlay system.	Standard porcine eye wet-lab training without AR assistance.	Wet-lab (Porcine Eye)	Cataract Phaco (Continuous Curvilinear Capsulorhexis CCC)
Nair, 2021 (50)	India	19	Residents & Fellows (Novice)	RCT	VR (HelpMeSee)	Practiced MSICS scleral tunnel construction on the HelpMeSee simulator preoperatively.	Received traditional wet-lab practice or observation preoperatively.	Live surgery	MSICS (Scleral Tunnel Construction)
Ng, 2018 (35)	China	19	Residents (Year 1-5)	Retrospective cohort	VR (Eyesi surgical)	Residents who had undergone VR simulation training.	Residents who had not undergone VR simulation training.	Questionnaire	Cataract Phaco (Cognitive/Learning barriers)
Peugnet, 1998 (51)	France	10	Residents (Novice)	RCT	VR (SOPHOCLE)	Training on the SOPHOCLE retinal photocoagulation simulator once weekly for 6 months.	Training on real patients under supervision once weekly for 6 months.	Live surgery	Panretinal Photocoagulation
Pokroy, 2013 (52)	USA	20	Residents (Novice)	Retrospective cohort	VR (Eyesi surgical)	Must complete at least 6 h of training on the Eyesi simulator within the first 18 months of residency.	Retrospective analysis of resident data prior to simulator introduction, receiving only traditional lectures and wet-lab training.	Live surgery	Cataract Phaco (Full procedure)
Saleh, 2013 (53)	UK	18	Trainees (ST1/Novice)	Single-arm	VR (Eyesi surgical)	Three consecutive attempts on 5 specific simulator tasks to assess repeatability.	N/A (Reliability and repeatability study).	VR simulator (Eyesi)	Cataract modules (Multiple tasks)
Saleh, 2015 (54)	UK	16	Trainees (Novice)	Single-arm Pre-post	VR (Eyesi surgical)	Completed at least 10 h of Eyesi curriculum, including at least 6 h of expert-supervised training.	N/A (Pre-course vs. Post-course comparison).	VR simulator (Eyesi)	Cataract modules (Full procedure sim)
Sankaran., 2022 (55)	India	10	Surgical trainees	Single-arm	VR (HelpMeSee)	Repeated practice of 6 key MSICS steps on the HelpMeSee simulator during a 1-month microsurgical rotation.	N/A (Retrospective observational study).	VR simulator (HelpMeSee)	MSICS (Key steps modules)
Staropoli, 2018 (18)	USA	22	Residents (PGY-3)	Retrospective cohort	VR (Eyesi surgical)	Mandatory completion of Eyesi CAT-A (Basic) and CAT-B (Advanced) modules before starting the cataract rotation.	Retrospective analysis of resident data prior to simulator introduction, receiving only lectures and early-year wet-lab training.	Live surgery	Cataract Phaco (Full procedure)
Sun, 2014 (25)	China	480	Medical students (Undergrad)	RCT	VR (Eyesi surgical)	Additional weekly surgical simulator operation training on top of traditional teaching.	Received only theoretical learning, surgical observation, and supervised stepwise surgical operations.	Clinical skills exam	Ophthalmic microsurgery (Basic skills)
Thomsen, 2017 (37)	Denmark	18	Surgeons (Novice to expert)	Single-arm Pre-post	VR (Eyesi surgical)	Repeated training on Eyesi until passing the proficiency test (score >600 in two consecutive courses).	N/A (Comparison of live surgeries before and after reaching proficiency).	Live surgery	Cataract Phaco (Full procedure)
Zhang, 2025 (16)	China	71	Optometry undergraduates	Non-RCT	VR (Screen-based system)	Interactive practice of the full process (history taking, examination, surgery) using a computer-based virtual simulation system.	Traditional offline practical training: including role-play history taking, surgical observation, and porcine eye wet-lab training.	Clinical Skills Exam	Cataract Phaco (Full procedure)

RCT, randomized controlled trial; PGY, postgraduate year; CCC, continuous curvilinear capsulorhexis; PCR, posterior capsule rupture; MSICS, manual small-incision cataract surgery; N/A, not applicable.

### Surgical complications

**Figure 2:**
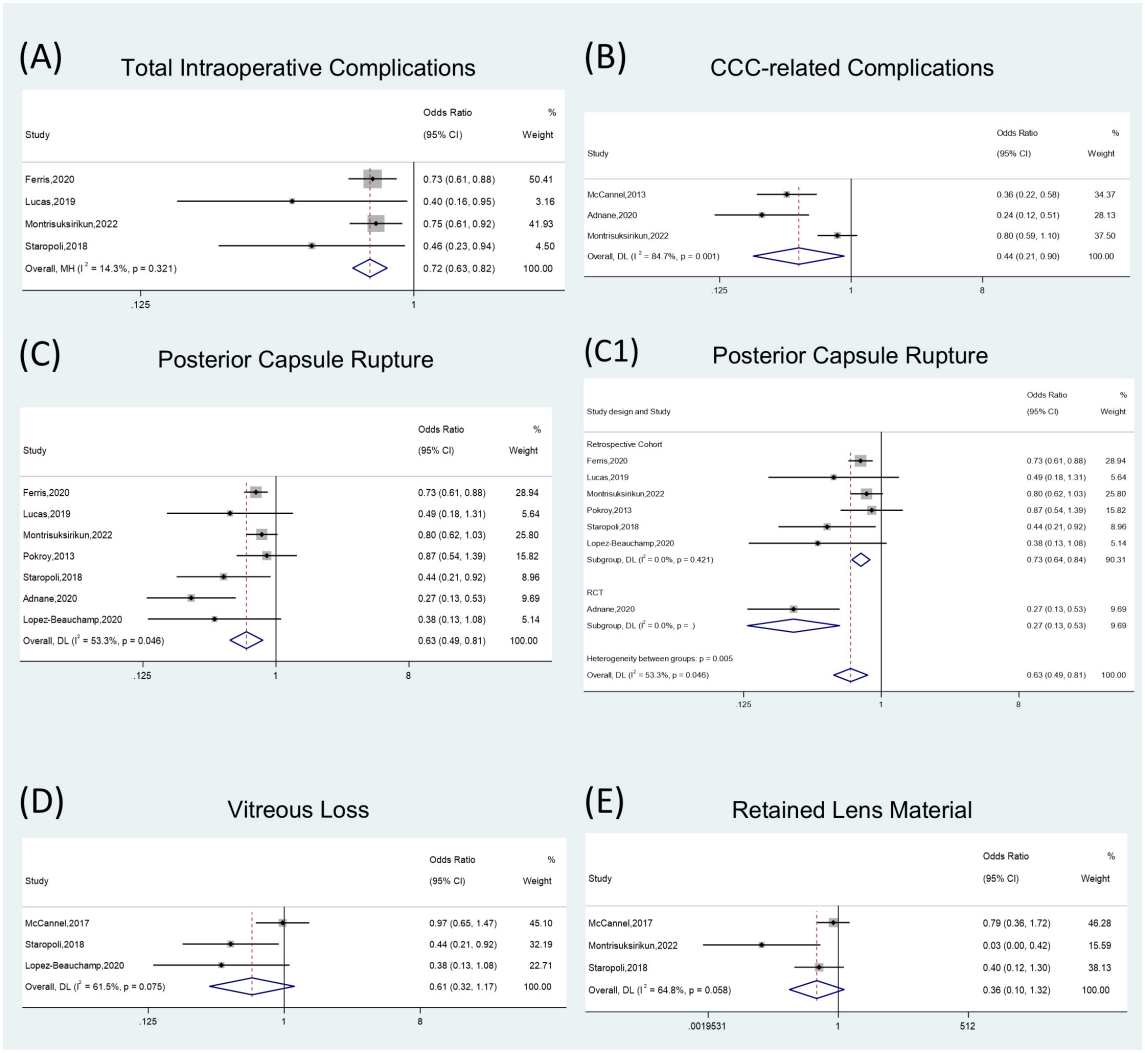
Forest plots illustrating the impact of XR training on intraoperative complications. **(A)** Total intraoperative complications. **(B)** Continuous curvilinear capsulorhexis (CCC)-related complications. **(C–C1)** Incidence of posterior capsule rupture (PCR) and subgroup analysis stratified by study design: randomized controlled trials vs. retrospective cohort studies. **(D)** Incidence of vitreous loss. **(E)** Incidence of retained lens material. CCC, continuous curvilinear capsulorhexis; PCR, posterior capsule rupture.

Four studies were included in the quantitative synthesis of intraoperative complications. Pooled results showed that, compared with controls, trainees who received simulation training had a lower overall intraoperative complication rate (OR = 0.72; 95% CI 0.63–0.82; *P* < 0.001). For specific complication subtypes, the rate of posterior capsule rupture was reduced (OR = 0.63; 95% CI 0.49–0.81; *P* < 0.001), and CCC-related complications were also reduced (OR = 0.44; 95% CI 0.21–0.90; *P* = 0.024). No statistically significant differences were found for vitreous loss (OR = 0.61; 95% CI 0.32–1.17; *P* = 0.136) or retained lens material (OR = 0.36; 95% CI 0.10–1.32; *P* = 0.122; Figure 2, Supplementary Figure 4).

Beyond cataract-related outcomes, Alwadani et al. (23) reported that during laser trabeculoplasty, the VR-trained group had a lower rate of missing the intended treatment location when performing procedures on real patients (*P* = 0.001).

For success-related outcomes, Le et al. (24) reported a higher procedure pass rate in the simulation group. Sun et al. (25) reported a higher proportion of trainees achieving scores >80 in the simulation group (*P* < 0.05). Ducloyer et al. (26) reported improvements in both overall and step-specific success rates after simulation training.

### Surgical performance scores

**Figure 3:**
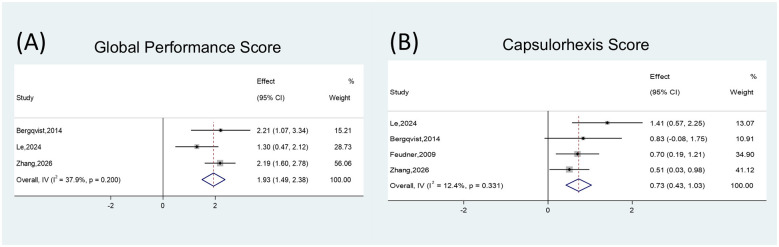
Forest plots illustrating the impact of XR training on surgical performance scores. **(A)** Global Performance Score. **(B)** Capsulorhexis score.

A pooled analysis of three studies showed that simulation training improved the overall surgical performance score (SMD = 1.93, 95% CI 1.49–2.38; *P* < 0.001). For step-specific outcomes, the continuous curvilinear capsulorhexis (CCC) score improved (SMD = 0.73, 95% CI 0.43–1.03; *P* < 0.001). Zhang et al. reported improved hydrodissection scores after simulation training (*P* = 0.0379) (16). Le and Hu further reported higher scores for cortical clean-up (irrigation/aspiration, I/A) and phacoemulsification-based nucleus management (*P* = 0.007 and *P* = 0.03, respectively) (24, 27). Beyond cataract surgery, Deuchler et al. (28) reported higher performance scores after a 20-min simulator “warm-up” before vitreoretinal surgery (*P* = 0.0302), and Deuchler et al. (29) reported higher assessment scores in the VR group for slit-lamp examination training (*P* < 0.001; Figure 3, Supplementary Figure 5).

### Procedure time and efficiency

**Figure 4:**
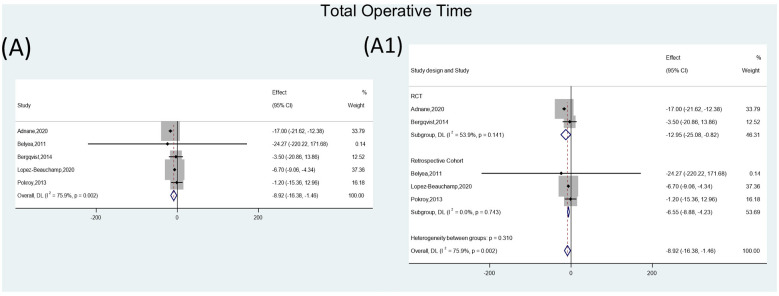
Forest plots illustrating the impact of XR training on total operative time. **(A)** Total operative time. **(A1)** Subgroup analysis stratified by study design: randomized controlled trials vs. retrospective cohort studies.

Five cataract surgery studies were pooled. Simulation training was associated with a shorter total operative time (WMD = −8.92; 95% CI −16.38 to −1.46; *P* = 0.019). Heterogeneity was high (*I*^2^ = 75.9%). After subgroup analyses by study type (RCTs vs. retrospective cohorts), heterogeneity decreased (*I*^2^ = 53.90% and 0.00%, respectively), and pooled effects remained statistically significant (*P* = 0.036 and *P* < 0.001, respectively). For step-specific time outcomes, Belyea et al. (30) reported shorter phacoemulsification time in the simulator group (*P* < 0.002). Gonzalez-Gonzalez et al. (31) found that after completing training modules, the time required to perform CCC decreased for both the dominant and non-dominant hands (*P* < 0.0001 and *P* < 0.02, respectively). Mathis et al. (32) reported that simulator training reduced the time needed to manage intraoperative challenges (Figure 4, Supplementary Figure 6).

Several studies also reported process measures related to efficiency. Adnane et al. (33) and Hu et al. (27) reported lower ultrasound energy use in the simulation group (*P* < 0.001 and *P* = 0.03, respectively). Gonzalez-Gonzalez et al. (31) also reported reduced instrument travel distance for both the dominant and non-dominant hands after completing training modules (*P* < 0.001).

### Self-efficacy and trainee experience

Seven studies reported subjective outcomes related to trainee experience. In terms of training satisfaction, Deuchler et al. (29) found that after slit-lamp simulation training, students reported significantly higher perceived improvement in relevant knowledge and skills. Daly et al. (34) reported that simulator training caused less frustration than wet-lab training. Regarding confidence, Gonzalez-Gonzalez et al. (31) reported that simulation training improved non-dominant hand dexterity and increased overall surgical confidence. Sun et al. (25) and Ng et al. (35) further reported that, compared with traditional training, Eyesi-based simulation increased confidence in performing complex steps and reduced perceived task difficulty. Regarding attitudes toward formal implementation, Staropoli et al. (18) and Gonzalez-Gonzalez et al. (31) reported that most trainees considered simulation training “highly valuable” and supported making it a mandatory component of residency training. For perceived realism, Hu et al. (27) found that although both groups considered training helpful and would recommend it, wet-lab training received higher realism scores than simulator training (*P* < 0.001). Daly et al. (34) reported that the two approaches had similar overall realism ratings.

### Other outcomes

Two studies specifically examined bimanual performance and consistently suggested greater benefit for the non-dominant hand. Gonzalez-Gonzalez et al. (31) reported improved Eyesi scores for both hands after training (dominant hand *P* = 0.048; non-dominant hand *P* = 0.0066), but the non-dominant hand showed a steeper learning curve and larger improvement. Eltanamly et al. (36) similarly reported significant improvement only for non-dominant hand dexterity (*P* < 0.001), while improvement in the dominant hand was not statistically significant (*P* = 0.12).

Regarding training seniority, Thomsen et al. suggested a “ceiling effect”: novices and intermediate surgeons showed significant improvement in real surgical performance after training, whereas differences were not significant among experienced surgeons (37). In addition, Le et al. (24) demonstrated cross-procedure skill transfer. Residents who achieved proficiency in phacoemulsification on Eyesi performed better on MSICS simulation tasks than controls without prior Phaco simulation experience, both in overall performance (*P* = 0.002) and pass rate (*P* = 0.018).

### Risk of bias, sensitivity analyses, and publication bias

All 32 included studies were appraised using RoB 2 and ROBINS-I tools. Among the 10 RCTs, 2 studies were judged to be at high risk of bias, 7 studies raised some concerns, and 1 study was rated as low risk. The primary issues identified were unclear allocation concealment in the randomization process and potential measurement bias arising from a lack of blinded outcome assessment. Among the 22 non-randomized studies, the majority (19 studies) were judged to have an overall moderate risk of bias. Three single-arm studies were rated as having serious risk of bias, primarily because the absence of concurrent controls made it difficult to distinguish improvements gained through simulation training from natural professional skill acquisition over time (Supplementary Figures 1, 2).

The GRADE evidence profile demonstrates that the Overall certainty of evidence for the primary safety outcomes of total intraoperative complications and posterior capsule rupture (PCR) was high. However, certainty of evidence was moderate for CCC-related complications, global surgical performance scores, and total operative time. Evidence for these outcomes was downgraded primarily due to the risk of bias in non-randomized study designs and inconsistency related to heterogeneity in training intensity across included studies (Supplementary Figure 3).

To assess potential publication bias, Egger's test was performed for outcomes with more than five studies. No significant publication bias was detected (all *P* > 0.05). Sensitivity analyses were conducted for all outcomes. After removing any single study, pooled effect sizes and statistical significance did not materially change, indicating good robustness of the meta-analysis (Supplementary Figures 4–6).

## Discussion

This systematic review and meta-analysis included 32 studies comprising 1,572 trainees. Overall, the findings indicate that, compared with conventional training, XR-based simulation training confers consistent advantages across multiple outcomes. Among pooled clinical safety outcomes, simulation training was associated with a lower overall risk of intraoperative complications (OR = 0.72). Reductions were particularly evident for posterior capsule rupture (OR = 0.63) and complications related to continuous curvilinear capsulorhexis (OR = 0.44), whereas no statistically significant differences were observed for outcomes such as vitreous prolapse and retained lens material. With respect to technical performance, simulation training significantly improved overall operative performance scores (SMD = 1.93) and enhanced scores for key steps including capsulorhexis and hydrodissection. Efficiency-related metrics further suggested that simulation training shortened total operative time (WMD = −8.92 min), and several studies additionally reported reductions in process measures such as ultrasound energy use and instrument path length. Beyond objective performance, most studies documented increased trainee satisfaction and confidence, although perceptions of “realism” remained heterogeneous when compared with wet-lab training. Collectively, these findings support the notion that XR simulation training not only improves measurable technical competence but may also yield benefits in patient safety and operative efficiency.

The observed reductions in complications and improvements in performance scores may be primarily attributable to the feedback mechanisms and autonomy inherent to simulation-based training. Unlike traditional apprenticeship models in clinical settings, simulators provide a standardized practice platform that enables trainees to perform repeated procedures in an environment free of patient-related risk. This iterative practice model, supported by immediate feedback, is underpinned by robust evidence: a systematic review of 109 studies reported that 47% identified educational feedback as a key component of simulation-based instruction, and 39% emphasized the importance of repetitive practice (38). Through this mechanism, trainees can identify errors and refine techniques within a controlled setting. In addition, automated guidance features can substantially alleviate instructional burden (7). Trainees may therefore engage in self-directed practice without continuous real-time supervision, which not only extends effective practice time but also allows them to adjust pacing according to individual learning trajectories, thereby facilitating skill acquisition.

The magnitude of benefit from simulation training appears inversely related to trainees' baseline experience; novices typically derive greater marginal gains from standardized practice. Thomsen et al. (37) demonstrated significant improvements in real-world surgical performance among novice and intermediate surgeons after training, whereas no statistically significant differences were observed among expert surgeons, suggesting a potential “ceiling effect” for simulation training. This pattern is particularly apparent in the development of bimanual skills. Gonzalez-Gonzalez et al. (31) found that improvements in EyeSi scores and the slope of the learning curve were significantly greater for the non-dominant hand than for the dominant hand, and Eltanamly et al. (36) further reported that only non-dominant-hand dexterity improved to a statistically significant degree. Ducloyer et al. (26) quantified this trend by analyzing learning curves, showing that novices exhibited an exponential decline in operative time and instrument travel distance early in training, followed by gradual stabilization. Evidence from laparoscopic and orthopedic surgery similarly corroborates this phenomenon, with experts requiring substantially fewer repetitions than novices to reach performance plateaus (39, 40). Taken together, these data suggest that XR technology is particularly well suited as an early intervention to help junior trainees overcome initial learning barriers. Moreover, given the substantial inter-individual variability in the time required to reach skill plateaus, simulation-based curricula should be proficiency-oriented, targeting predefined competency benchmarks, rather than predicated solely on completing a fixed number of repetitions or hours.

Prior research on simulators and XR in ophthalmic training has yielded inconsistent conclusions regarding operative time. Although most studies suggest reductions in total time and step-specific durations, some have not demonstrated statistically significant differences. Carr et al. (20) similarly reported that pooled differences in “task completion time” were not significant and were characterized by very high heterogeneity. To address this issue, the present meta-analysis applied stringent inclusion criteria, pooling only data pertaining to “complete procedures” and excluding step-level time measures. The results showed a significant reduction in total operative time in the simulation group (WMD = −8.92 min). While overall heterogeneity remained substantial (*I*^2^ = 75.9%), stratification by study design markedly reduced heterogeneity within subgroups (0.0% and 53.9%, respectively), supporting the robustness of the time-saving effect under specific controlled conditions. Mechanistically, the reduction in operative time is unlikely to reflect mere acceleration of movements; rather, it plausibly represents a natural consequence of improved proficiency. Empirical evidence indicates that simulation training enhances the stability of hand–eye coordination and optimizes instrument trajectories, objectively reflected by shorter instrument path length and lower cumulative dissipated energy (CDE), which in turn suggests fewer inefficient maneuvers and smoother procedural flow (26, 27, 30, 31, 33). Accordingly, shorter operative time should be interpreted as a composite manifestation of improved efficiency rather than an explicit training target; particularly in high-risk steps, speed should never be prioritized at the expense of safety.

In addition to objective technical performance, trainees' self-efficacy and satisfaction constitute important dimensions of training evaluation. Available evidence suggests that simulation training can significantly increase trainee confidence and help alleviate anxiety among novices. Nonetheless, the absence of authentic haptic feedback remains a major limitation of current simulators (7). Because simulators cannot fully replicate the resistance and mechanical properties of real tissues, comparative studies have reported significantly higher realism ratings for wet-lab training than for simulators (27). To address this limitation, emerging XR technologies are increasingly focused on enhancing the authenticity of haptic interaction (41). Platforms such as HelpMeSee integrate physical haptic feedback systems, enabling trainees to perceive tissue tension in tasks such as incision creation and suturing (12). Similarly, systems such as Bionic-Eye incorporate force sensors to monitor and quantify surgical force in real time (42). These advances are progressively mitigating the tactile limitations of XR-based approaches and improving perceived realism. Nevertheless, despite ongoing improvements, the most feasible near-term pathway remains a hybrid curriculum that combines simulators and wet laboratories: simulators can be used for standardized procedural training, while wet labs provide authentic tissue handling experiences, thereby leveraging complementary strengths.

Beyond skill acquisition, XR simulators may also serve as tools for preoperative warm-up and for bridging learning across procedures. One study in vitreoretinal surgery-related tasks reported higher intraoperative technical performance scores following approximately 20 min of preoperative simulator practice (28), suggesting that brief, targeted rehearsal may improve operative readiness. However, current evidence is largely derived from a single study, and the applicable populations and optimal duration require further validation. In parallel, Le et al. (24) described cross-procedure skill transfer, whereby mastery of key steps in one simulated procedure facilitated early learning in another related procedure. This effect was primarily observed in steps with similar motor patterns, but was less apparent for procedure-specific steps, implying that the extent of transfer is contingent on step-level similarity. These findings provide a rationale for optimizing training pathways by using simulators to strengthen foundational microsurgical skills shared across multiple procedures and by positioning simulation as a transitional bridge between different procedural trainings.

In summary, the present findings indicate that XR simulation training is positively associated with improved surgical skills, a lower risk of intraoperative complications, greater operative efficiency, and stronger trainee self-efficacy. A key strength of this study lies in its comprehensive identification of available primary studies. In comparison with prior reviews, it also incorporated additional quantitative syntheses of process measures and subgroup analyses, thereby providing more granular evidence. Another notable strength is that, beyond cataract surgery, the review extended to XR training developments in other subspecialties, such as vitreoretinal surgery, thus offering a broader evidentiary basis for the application of this technology.

Nevertheless, the current body of evidence has notable limitations that delineate priorities for future research. Firstly, quantitative syntheses for several outcomes were based on a limited number of studies and relatively small total sample sizes per outcome, and heterogeneity in curriculum design, assessment standards, and outcome definitions may have reduced precision. More standardized, multicenter randomized controlled trials are therefore needed to improve reliability and reduce heterogeneity. Secondly, most included studies evaluated VR-based cataract simulators, particularly Eyesi, whereas AR-based training and non-cataract procedures were represented by only a few studies (12). Accordingly, the generalizability of the present findings to AR platforms and other ophthalmic subspecialties remains uncertain. Thirdly, Mathis et al. (32) reported that marked improvements in simulation scores were associated with approximately 14 h of cumulative training; however, most existing studies implemented substantially shorter training durations, potentially underestimating the true efficacy of simulation-based training. Future studies should adopt more standardized training “doses” and protocols. Fourthly, subjective outcomes such as confidence, satisfaction, frustration, and perceived realism were often assessed using non-uniform or study-specific instruments rather than standardized scales, which limits cross-study comparability. Finally, the literature has largely focused on technical skills, with only Zhang et al. (16) evaluating clinical reasoning or diagnostic ability. Given the centrality of clinical decision-making and patient–clinician communication in surgical practice, future evaluation frameworks should extend to non-technical skills (NTS).

## Conclusion

This updated meta-analysis of 32 studies (1,572 trainees) demonstrates that XR simulation training lowers overall intraoperative complications (OR 0.72), reduces posterior capsule rupture (OR 0.63) and CCC-related complications (OR 0.44), improves global surgical performance (SMD 1.93) and CCC scores (SMD 0.73), shortens total operative time (WMD −8.92 min), and generally increases trainee confidence and satisfaction. XR simulation appears to complement conventional operating-room and wet-lab training. Among the different approaches, evidence was strongest for VR cataract simulation, particularly Eyesi-based curricula overall, whereas AR systems and non-cataract procedures remain understudied. Given these gains, XR simulation should be incorporated into ophthalmology curricula. Future large, well-designed multicenter RCTs with standardized outcomes and long-term follow-up are warranted to optimize training dose and support broader implementation.

## Data Availability

The datasets presented in this study can be found in online repositories. The names of the repository/repositories and accession number(s) can be found in the article/Supplementary material.
