# Supplementary material for: Impact of extended reality (XR) simulation on ophthalmology training outcomes: an updated systematic review and meta-analysis
Source: Front Med (Lausanne). 2026 May 1;13:1823359. doi: 10.3389/fmed.2026.1823359 (PMC13175883; doi:10.3389/fmed.2026.1823359)
Supplement: Supplementary file 1 [file Data_Sheet_1.docx]

**Appendix 1. Representative search strategy used in PubMed.**

#1: (Ophthalmologic Surgical Procedures[Mesh] OR Blepharoplasty[Mesh] OR Dacryocystorhinostomy[Mesh] OR Eye Enucleation[Mesh] OR Eye Evisceration[Mesh] OR Filtering Surgery[Mesh] OR Iridectomy[Mesh] OR Light Coagulation[Mesh] OR Orbit Evisceration[Mesh] OR Refractive Surgical Procedures[Mesh] OR Scleral Buckling[Mesh] OR Vitrectomy[Mesh] OR Vitreoretinal Surgery[Mesh] OR Sclerostomy[Mesh] OR Trabeculectomy[Mesh] OR Cataract Extraction[Mesh] OR Corneal Surgery, Laser[Mesh] OR Corneal Transplantation[Mesh] OR Keratotomy, Radial[Mesh] OR Lens Implantation, Intraocular[Mesh] OR Posterior Capsulotomy[Mesh] OR Scleroplasty[Mesh] OR "Blepharoplasties"[Title/Abstract] OR "Dacryocystorhinostomies"[Title/Abstract] OR "Dacryocystostomy"[Title/Abstract] OR "Dacryocystostomies"[Title/Abstract] OR "Enucleation, Eye"[Title/Abstract] OR "Eye Enucleations"[Title/Abstract] OR "Enucleation Technique"[Title/Abstract] OR "Enucleation Techniques"[Title/Abstract] OR "Enucleation Surgery"[Title/Abstract] OR "Enucleation Surgeries"[Title/Abstract] OR "Surgery, Enucleation"[Title/Abstract] OR "Evisceration, Eye"[Title/Abstract] OR "Eviscerations, Eye"[Title/Abstract] OR "Eye Eviscerations"[Title/Abstract] OR "Surgery, Filtering"[Title/Abstract] OR "Filtration Surgery"[Title/Abstract] OR "Surgery, Filtration"[Title/Abstract] OR "Iridectomies"[Title/Abstract] OR "Corectomy"[Title/Abstract] OR "Corectomies"[Title/Abstract] OR "Coagulation, Light"[Title/Abstract] OR "Coagulations, Light"[Title/Abstract] OR "Light Coagulations"[Title/Abstract] OR "Photocoagulation"[Title/Abstract] OR "Photocoagulations"[Title/Abstract] OR "Evisceration, Orbit"[Title/Abstract] OR "Eviscerations, Orbit"[Title/Abstract] OR "Orbit Eviscerations"[Title/Abstract] OR "Orbital Evisceration"[Title/Abstract] OR "Evisceration, Orbital"[Title/Abstract] OR "Eviscerations, Orbital"[Title/Abstract] OR "Orbital Eviscerations"[Title/Abstract] OR "Procedure, Refractive Surgical"[Title/Abstract] OR "Procedures, Refractive Surgical"[Title/Abstract] OR "Refractive Surgical Procedure"[Title/Abstract] OR "Surgical Procedure, Refractive"[Title/Abstract] OR "Surgical Procedures, Refractive"[Title/Abstract] OR "Refractive Surgery"[Title/Abstract] OR "Refractive Surgeries"[Title/Abstract] OR "Surgeries, Refractive"[Title/Abstract] OR "Surgery, Refractive"[Title/Abstract] OR "Keratorefractive Surgical Procedures"[Title/Abstract] OR "Keratorefractive Surgical Procedure"[Title/Abstract] OR "Procedure, Keratorefractive Surgical"[Title/Abstract] OR "Procedures, Keratorefractive Surgical"[Title/Abstract] OR "Surgical Procedure, Keratorefractive"[Title/Abstract] OR "Surgical Procedures, Keratorefractive"[Title/Abstract] OR "Buckling, Scleral"[Title/Abstract] OR "Bucklings, Scleral"[Title/Abstract] OR "Scleral Bucklings"[Title/Abstract] OR "Vitrectomies"[Title/Abstract] OR "Surgeries, Vitreoretinal"[Title/Abstract] OR "Surgery, Vitreoretinal"[Title/Abstract] OR "Vitreoretinal Surgeries"[Title/Abstract] OR "Sclerostomies"[Title/Abstract] OR "Trabeculectomies"[Title/Abstract] OR "Trabeculoplasty"[Title/Abstract] OR "Trabeculoplasties"[Title/Abstract] OR "Goniotomy"[Title/Abstract] OR "Goniotomies"[Title/Abstract] OR "Trabeculotomy"[Title/Abstract] OR "Trabeculotomies"[Title/Abstract] OR "Cataract Extractions"[Title/Abstract] OR "Extraction, Cataract"[Title/Abstract] OR "Extractions, Cataract"[Title/Abstract] OR "Enzymatic Zonulolysis"[Title/Abstract] OR "Enzymatic Zonulolyses"[Title/Abstract] OR "Zonulolyses, Enzymatic"[Title/Abstract] OR "Zonulolysis, Enzymatic"[Title/Abstract] OR "Phakectomy"[Title/Abstract] OR "Phakectomies"[Title/Abstract] OR "Corneal Surgeries, Laser"[Title/Abstract] OR "Laser Corneal Surgeries"[Title/Abstract] OR "Surgeries, Laser Corneal"[Title/Abstract] OR "Surgery, Laser Corneal"[Title/Abstract] OR "Laser Corneal Surgery"[Title/Abstract] OR "Keratectomy, Laser"[Title/Abstract] OR "Keratectomies, Laser"[Title/Abstract] OR "Laser Keratectomies"[Title/Abstract] OR "Photokeratectomy"[Title/Abstract] OR "Photokeratectomies"[Title/Abstract] OR "Laser Keratectomy"[Title/Abstract] OR "Transplantation, Corneal"[Title/Abstract] OR "Corneal Transplantations"[Title/Abstract] OR "Transplantations, Corneal"[Title/Abstract] OR "Grafting, Corneal"[Title/Abstract] OR "Corneal Grafting"[Title/Abstract] OR "Corneal Graftings"[Title/Abstract] OR "Graftings, Corneal"[Title/Abstract] OR "Keratoplasty"[Title/Abstract] OR "Keratoplasties"[Title/Abstract] OR "Cornea Transplantation"[Title/Abstract] OR "Cornea Transplantations"[Title/Abstract] OR "Transplantations, Cornea"[Title/Abstract] OR "Transplantation, Cornea"[Title/Abstract] OR "Keratoplasty, Lamellar"[Title/Abstract] OR "Keratoplasties, Lamellar"[Title/Abstract] OR "Lamellar Keratoplasties"[Title/Abstract] OR "Lamellar Keratoplasty"[Title/Abstract] OR "Radial Keratotomy"[Title/Abstract] OR "Keratotomies, Radial"[Title/Abstract] OR "Radial Keratotomies"[Title/Abstract] OR "Implantation, Intraocular Lens"[Title/Abstract] OR "Implantations, Intraocular Lens"[Title/Abstract] OR "Intraocular Lens Implantation"[Title/Abstract] OR "Intraocular Lens Implantations"[Title/Abstract] OR "Lens Implantations, Intraocular"[Title/Abstract] OR "Capsulotomies, Posterior"[Title/Abstract] OR "Capsulotomy, Posterior"[Title/Abstract] OR "Posterior Capsulotomies"[Title/Abstract] OR "Posterior Capsulotomy, Laser"[Title/Abstract] OR "Capsulotomies, Laser Posterior"[Title/Abstract] OR "Capsulotomy, Laser Posterior"[Title/Abstract] OR "Laser Posterior Capsulotomies"[Title/Abstract] OR "Laser Posterior Capsulotomy"[Title/Abstract] OR "Posterior Capsulotomies, Laser"[Title/Abstract] OR "Scleroplasties"[Title/Abstract])

#2: (Computer Simulation[Mesh] OR Augmented Reality[Mesh] OR Virtual Reality[Mesh] OR Haptic Technology[Mesh] OR Avatar[Mesh] OR User-Computer Interface[Mesh] OR Ambient Intelligence[Mesh] OR Simulation Training[Mesh] OR "Augmented Realities"[Title/Abstract] OR "Realities, Augmented"[Title/Abstract] OR "Reality, Augmented"[Title/Abstract] OR "Mixed Reality"[Title/Abstract] OR "Mixed Realities"[Title/Abstract] OR "Realities, Mixed"[Title/Abstract] OR "Reality, Mixed"[Title/Abstract] OR "Reality, Virtual"[Title/Abstract] OR "Virtual Reality, Educational"[Title/Abstract] OR "Educational Virtual Realities"[Title/Abstract] OR "Educational Virtual Reality"[Title/Abstract] OR "Reality, Educational Virtual"[Title/Abstract] OR "Virtual Realities, Educational"[Title/Abstract] OR "Virtual Reality, Instructional"[Title/Abstract] OR "Instructional Virtual Realities"[Title/Abstract] OR "Instructional Virtual Reality"[Title/Abstract] OR "Realities, Instructional Virtual"[Title/Abstract] OR "Reality, Instructional Virtual"[Title/Abstract] OR "Virtual Realities, Instructional"[Title/Abstract] OR "Haptic Technologies"[Title/Abstract] OR "Technologies, Haptic"[Title/Abstract] OR "Technology, Haptic"[Title/Abstract] OR "Touch Feedback Technology"[Title/Abstract] OR "Technologies, Touch Feedback"[Title/Abstract] OR "Technology, Touch Feedback"[Title/Abstract] OR "Touch Feedback Technologies"[Title/Abstract] OR "Haptic Feedback Technology"[Title/Abstract] OR "Haptic Feedback Technologies"[Title/Abstract] OR "Technologies, Haptic Feedback"[Title/Abstract] OR "Technology, Haptic Feedback"[Title/Abstract] OR "Haptic Communication Technology"[Title/Abstract] OR "Communication Technologies, Haptic"[Title/Abstract] OR "Communication Technology, Haptic"[Title/Abstract] OR "Haptic Communication Technologies"[Title/Abstract] OR "Technologies, Haptic Communication"[Title/Abstract] OR "Technology, Haptic Communication"[Title/Abstract] OR "Haptic Perception Technology"[Title/Abstract] OR "Haptic Perception Technologies"[Title/Abstract] OR "Perception Technologies, Haptic"[Title/Abstract] OR "Perception Technology, Haptic"[Title/Abstract] OR "Technologies, Haptic Perception"[Title/Abstract] OR "Technology, Haptic Perception"[Title/Abstract] OR "Haptics"[Title/Abstract] OR "Avatars"[Title/Abstract] OR "Humanoid Avatar"[Title/Abstract] OR "Avatar, Humanoid"[Title/Abstract] OR "Humanoid Avatars"[Title/Abstract] OR "Computer Simulations"[Title/Abstract] OR "Simulation, Computer"[Title/Abstract] OR "Simulations, Computer"[Title/Abstract] OR "Computer Models"[Title/Abstract] OR "Computer Model"[Title/Abstract] OR "Model, Computer"[Title/Abstract] OR "In silico Models"[Title/Abstract] OR "In silico Model"[Title/Abstract] OR "Model, In silico"[Title/Abstract] OR "In silico Simulation"[Title/Abstract] OR "Simulation, In silico"[Title/Abstract] OR "Computerized Models"[Title/Abstract] OR "Computerized Model"[Title/Abstract] OR "Model, Computerized"[Title/Abstract] OR "Models, Computer"[Title/Abstract] OR "Computational Modelling"[Title/Abstract] OR "Modelling, Computational"[Title/Abstract] OR "In silico Modeling"[Title/Abstract] OR "Modeling, In silico"[Title/Abstract] OR "Computational Modeling"[Title/Abstract] OR "Modeling, Computational"[Title/Abstract] OR "Interfaces, User-Computer"[Title/Abstract] OR "Interface, User-Computer"[Title/Abstract] OR "User-Computer Interfaces"[Title/Abstract] OR "User Computer Interface"[Title/Abstract] OR "Interfaces, User Computer"[Title/Abstract] OR "User Computer Interfaces"[Title/Abstract] OR "Interface, User Computer"[Title/Abstract] OR "Virtual Systems"[Title/Abstract] OR "Systems, Virtual"[Title/Abstract] OR "System, Virtual"[Title/Abstract] OR "Virtual System"[Title/Abstract] OR "Ambient Intelligences"[Title/Abstract] OR "Intelligence, Ambient"[Title/Abstract] OR "Intelligences, Ambient"[Title/Abstract] OR "Ambient Assisted Living"[Title/Abstract] OR "Ambient Assisted Livings"[Title/Abstract] OR "Assisted Living, Ambient"[Title/Abstract] OR "Assisted Livings, Ambient"[Title/Abstract] OR "Living, Ambient Assisted"[Title/Abstract] OR "Livings, Ambient Assisted"[Title/Abstract] OR "Ambient-Assisted Living"[Title/Abstract] OR "Ambient-Assisted Livings"[Title/Abstract] OR "Living, Ambient-Assisted"[Title/Abstract] OR "Livings, Ambient-Assisted"[Title/Abstract] OR "Training, Simulation"[Title/Abstract] OR "Interactive Learning"[Title/Abstract] OR "Learning, Interactive"[Title/Abstract] OR "Eyesi"[Title/Abstract])

#3: (Patient Simulation[Mesh] OR Computer-Assisted Instruction[Mesh] OR Education, Medical[Mesh] OR Education, Medical, Undergraduate[Mesh] OR Education, Medical, Graduate[Mesh] OR Education, Medical, Continuing[Mesh] OR Internship and Residency[Mesh] OR Students, Medical[Mesh] OR Curriculum[Mesh] OR Clinical Competence[Mesh] OR Preceptorship[Mesh] OR "Patient Simulations"[Title/Abstract] OR "Simulation, Patient"[Title/Abstract] OR "Simulations, Patient"[Title/Abstract] OR "Computer Assisted Instruction"[Title/Abstract] OR "Computer-Assisted Instructions"[Title/Abstract] OR "Instruction, Computer-Assisted"[Title/Abstract] OR "Instructions, Computer-Assisted"[Title/Abstract] OR "Self-Instruction Programs, Computerized"[Title/Abstract] OR "Computerized Self-Instruction Program"[Title/Abstract] OR "Computerized Self-Instruction Programs"[Title/Abstract] OR "Program, Computerized Self-Instruction"[Title/Abstract] OR "Programs, Computerized Self-Instruction"[Title/Abstract] OR "Self-Instruction Program, Computerized"[Title/Abstract] OR "Self Instruction Programs, Computerized"[Title/Abstract] OR "Programmed Instruction, Computerized"[Title/Abstract] OR "Computerized Programmed Instruction"[Title/Abstract] OR "Instruction, Computerized Programmed"[Title/Abstract] OR "Medical Education"[Title/Abstract] OR "Education, Undergraduate Medical"[Title/Abstract] OR "Medical Education, Undergraduate"[Title/Abstract] OR "Undergraduate Medical Education"[Title/Abstract] OR "Graduate Medical Education"[Title/Abstract] OR "Medical Education, Graduate"[Title/Abstract] OR "Education, Graduate Medical"[Title/Abstract] OR "Continuing Medical Education"[Title/Abstract] OR "Education, Continuing Medical"[Title/Abstract] OR "Medical Education, Continuing"[Title/Abstract] OR "Residency and Internship"[Title/Abstract] OR "Internship, Medical"[Title/Abstract] OR "Internships, Medical"[Title/Abstract] OR "Medical Internship"[Title/Abstract] OR "Medical Internships"[Title/Abstract] OR "Internship"[Title/Abstract] OR "House Staff"[Title/Abstract] OR "Staff, House"[Title/Abstract] OR "Residency"[Title/Abstract] OR "Residencies"[Title/Abstract] OR "Medical Residencies"[Title/Abstract] OR "Medical Residency"[Title/Abstract] OR "Residencies, Medical"[Title/Abstract] OR "Residency, Medical"[Title/Abstract] OR "Medical Student"[Title/Abstract] OR "Medical Students"[Title/Abstract] OR "Student, Medical"[Title/Abstract] OR "Curricula"[Title/Abstract] OR "Short-Term Courses"[Title/Abstract] OR "Course, Short-Term"[Title/Abstract] OR "Courses, Short-Term"[Title/Abstract] OR "Short-Term Course"[Title/Abstract] OR "Short Term Courses"[Title/Abstract] OR "Clinical Competency"[Title/Abstract] OR "Clinical Competencies"[Title/Abstract] OR "Competencies, Clinical"[Title/Abstract] OR "Competency, Clinical"[Title/Abstract] OR "Competence, Clinical"[Title/Abstract] OR "Clinical Skill"[Title/Abstract] OR "Clinical Skills"[Title/Abstract] OR "Skill, Clinical"[Title/Abstract] OR "Skills, Clinical"[Title/Abstract] OR "Clinical Practicum"[Title/Abstract] OR "Practicum, Clinical"[Title/Abstract] OR "Practicums, Clinical"[Title/Abstract] OR "Field Work, Medical"[Title/Abstract] OR "Medical Field Work"[Title/Abstract] OR "Work, Medical Field"[Title/Abstract] OR "Field Study, Medical"[Title/Abstract] OR "Field Studies, Medical"[Title/Abstract] OR "Medical Field Studies"[Title/Abstract] OR "Medical Field Study"[Title/Abstract] OR "Studies, Medical Field"[Title/Abstract] OR "Study, Medical Field"[Title/Abstract] OR "Field Training, Medical"[Title/Abstract] OR "Medical Field Training"[Title/Abstract] OR "Training, Medical Field"[Title/Abstract] OR "Clinical Supervision"[Title/Abstract] OR "Clinical Supervisions"[Title/Abstract] OR "Supervision, Clinical"[Title/Abstract] OR "Supervisions, Clinical"[Title/Abstract] OR "Clinical Mentoring"[Title/Abstract] OR "Clinical Mentorings"[Title/Abstract] OR "Mentoring, Clinical"[Title/Abstract] OR "Mentorings, Clinical"[Title/Abstract])

#4: #1 AND #2 AND #3

**Supplementary Table S1. Summary of search results from electronic databases.**

| Database | Coverage (timespan) | Limits/filters | Records retrieved (n) |
| --- | --- | --- | --- |
| Web of Science (WoS) | Inception to 2026-02-06 | None | 222 |
| Cochrane Library | Inception to 2026-02-06 | None | 45 |
| Embase | Inception to 2026-02-06 | None | 259 |
| PubMed | Inception to 2026-02-06 | None | 158 |
| Total |  |  | 684 |

**Supplementary Table S2. Baseline characteristics of the included studies.**

| Category | N | % |
| --- | --- | --- |
| Study Design |  |  |
| RCT | 10 | 31.30% |
| Retrospective Cohort | 10 | 31.30% |
| Single-arm (Pre-post) | 8 | 25.00% |
| Non-RCT | 4 | 12.50% |
| Simulation Modality |  |  |
| Eyesi Surgical (VR) | 24 | 75.00% |
| HelpMeSee (VR) | 3 | 9.40% |
| Other VR Simulators | 3 | 9.40% |
| Eyesi Slit Lamp (VR) | 1 | 3.10% |
| Augmented Reality (AR) | 1 | 3.10% |
| Target Procedure |  |  |
| Cataract Phaco | 24 | 75.00% |
| Cataract MSICS | 3 | 9.40% |
| Laser Trabeculoplasty | 1 | 6.30% |
| Ophthalmic Microsurgery | 1 | 9.40% |
| Panretinal Photocoagulation | 1 | 3.13% |
| Pars Plana Vitrectomy | 1 | 3.13% |
| Slit Lamp Examination | 1 | 3.13% |
| Assessment Platform |  |  |
| Live Surgery | 15 | 46.90% |
| VR Simulator | 10 | 31.30% |
| Wet-lab / Dry-lab | 3 | 9.40% |
| Clinical Exam | 3 | 9.40% |
| Questionnaire | 1 | 3.10% |
| Population |  |  |
| Ophthalmology Residents | 21 | 65.60% |
| Medical Students | 6 | 18.80% |
| ophthalmologists, fellows, or mixed | 5 | 15.63% |

**Supplementary Table S3. Summary of pooled effect sizes for all outcome measures.**

| Outcome | | Number of studies | Effect Size*** | 95%CI | I² | P-Value* | Egger's Test |
| --- | --- | --- | --- | --- | --- | --- | --- |
| Intraoperative Complications | Total Intraoperative Complications | 4 | 0.72 | 0.63-0.82 | 14.3% | ＜0.001 | —** |
|  | Posterior Capsule Rupture (PCR) | 7 | 0.63 | 0.49-0.81 | 53.3% | ＜0.001 | 0.077 |
|  | Vitreous Loss | 3 | 0.61 | 0.32-1.17 | 61.5% | 0.136 | — |
|  | CCC-related Complications | 3 | 0.44 | 0.21-0.90 | 84.7% | 0.024 | — |
|  | Retained Lens Material | 3 | 0.36 | 0.10-1.32 | 64.8% | 0.122 | — |
| Surgical Competence Scores | Global Performance Score | 3 | 1.93 | 1.49-2.38 | 37.9% | ＜0.001 | — |
|  | Capsulorhexis Score | 4 | 0.73 | 0.43-1.03 | 12.4% | ＜0.001 | — |
| Operative Time | Total Operative Time | 5 | -8.92 | -16.38--1.46 | 75.9% | 0.019 | 0.891 |

Note: *P values are for the test of overall effect (H₀: effect size = 0). Heterogeneity P values are not shown. **Egger’s test was not performed when the number of studies was less than 5 (n < 5). ***Effect sizes are reported as Odds Ratio (OR) for complications, Mean Difference (MD) for operative time, and Standardized Mean Difference (SMD) for competence scores.

**Supplementary Table S4. Results of subgroup analyses.**

| Outcome | Subgroup | | Number of studies | Effect Size** | 95%CI | I² | P-Value* |
| --- | --- | --- | --- | --- | --- | --- | --- |
|  | Type | Group |  |  |  |  |  |
| Posterior Capsule Rupture (PCR) | Study design | Retrospective Cohort | 6 | 0.73 | 0.64-0.84 | 0.00% | ＜0.001 |
|  |  | RCT | 1 | 0.27 | 0.13-0.53 | NA (k=1) | ＜0.001 |
| Operative Time | Study design | Retrospective Cohort | 3 | -6.55 | -8.88--4.23 | 0.00% | ＜0.001 |
|  |  | RCT | 2 | -12.95 | -25.08--0.82 | 53.90% | 0.036 |

Note: *P values are for the test of overall effect (H₀: effect size = 0). Heterogeneity P values are not shown. **Effect sizes are reported as Odds Ratio (OR) for complications, Mean Difference (MD) for operative time, and Standardized Mean Difference (SMD) for competence scores.


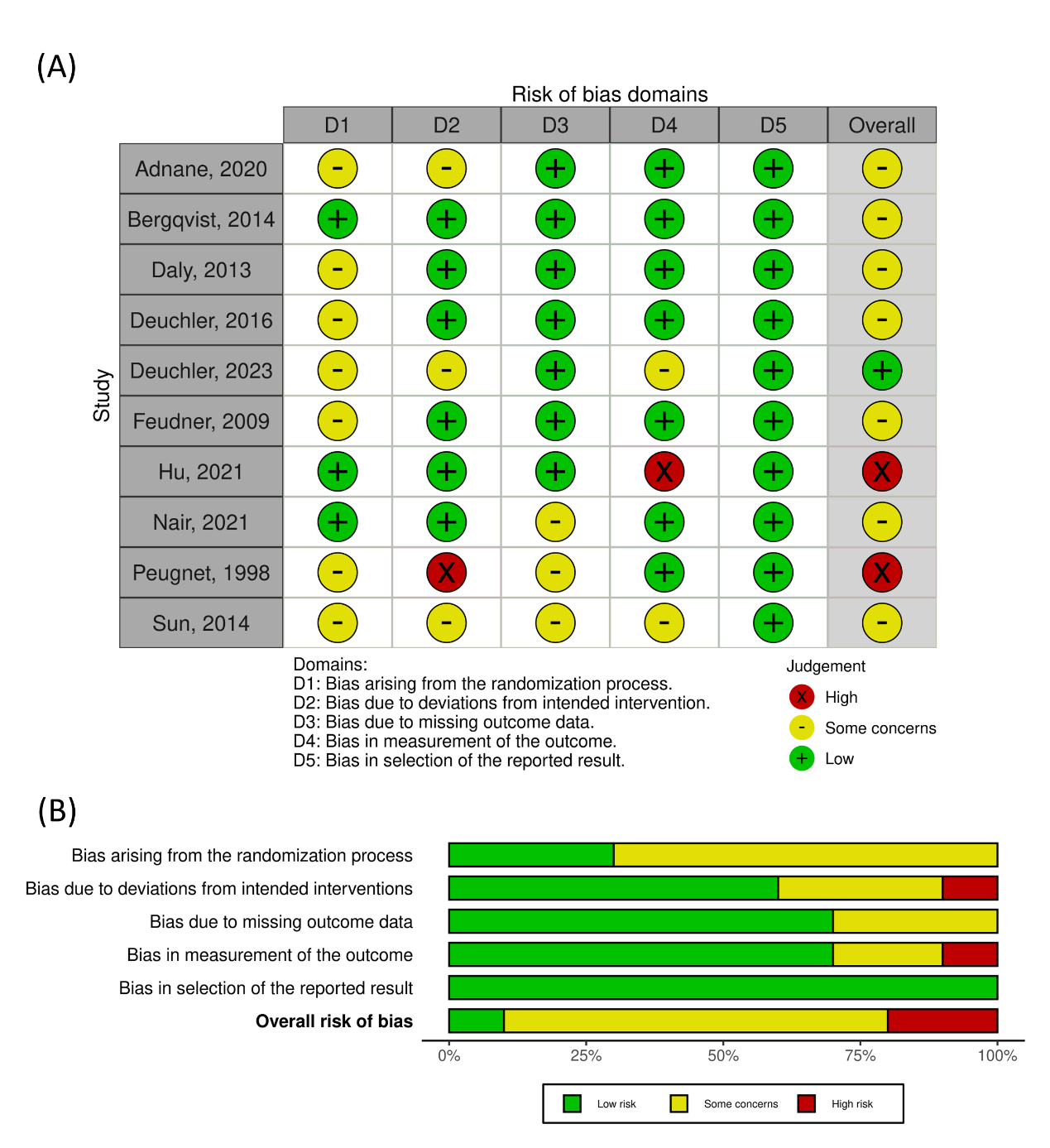


**Supplementary Figure 1. Risk of bias assessment for randomized controlled trials using the RoB 2 tool.**

**(A)** Cochrane Risk of Bias 2.0 tool analysis for individual studies. **(B)** Cochrane Risk of Bias 2.0 tool outcomes grouped across all included trials.


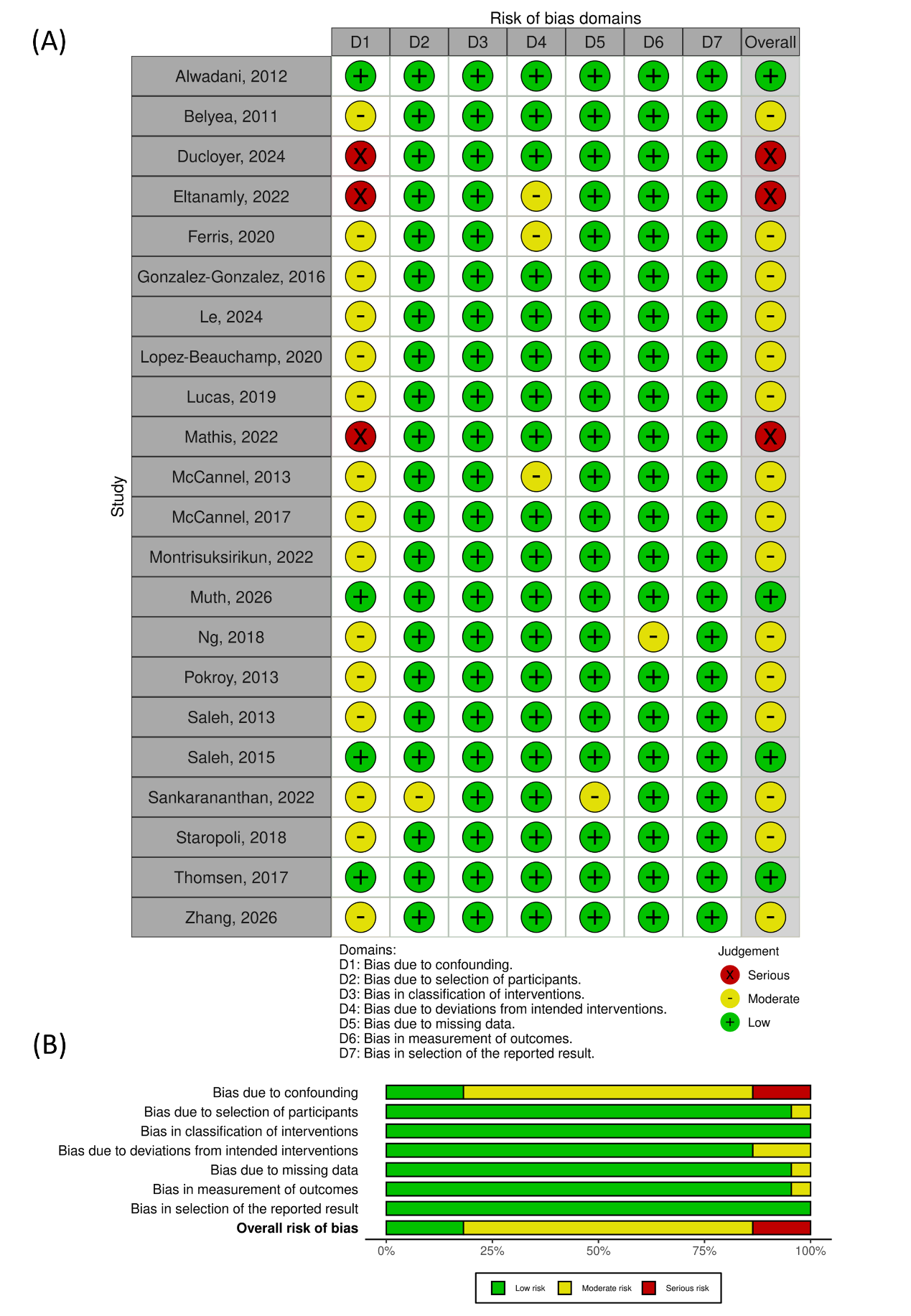


**Supplementary Figure 2. Risk of bias assessment for non-randomized studies using the ROBINS-I tool.**

**(A)** ROBINS-I tool analysis for individual observational studies. **(B)** ROBINS-I tool outcomes grouped across all included studies.


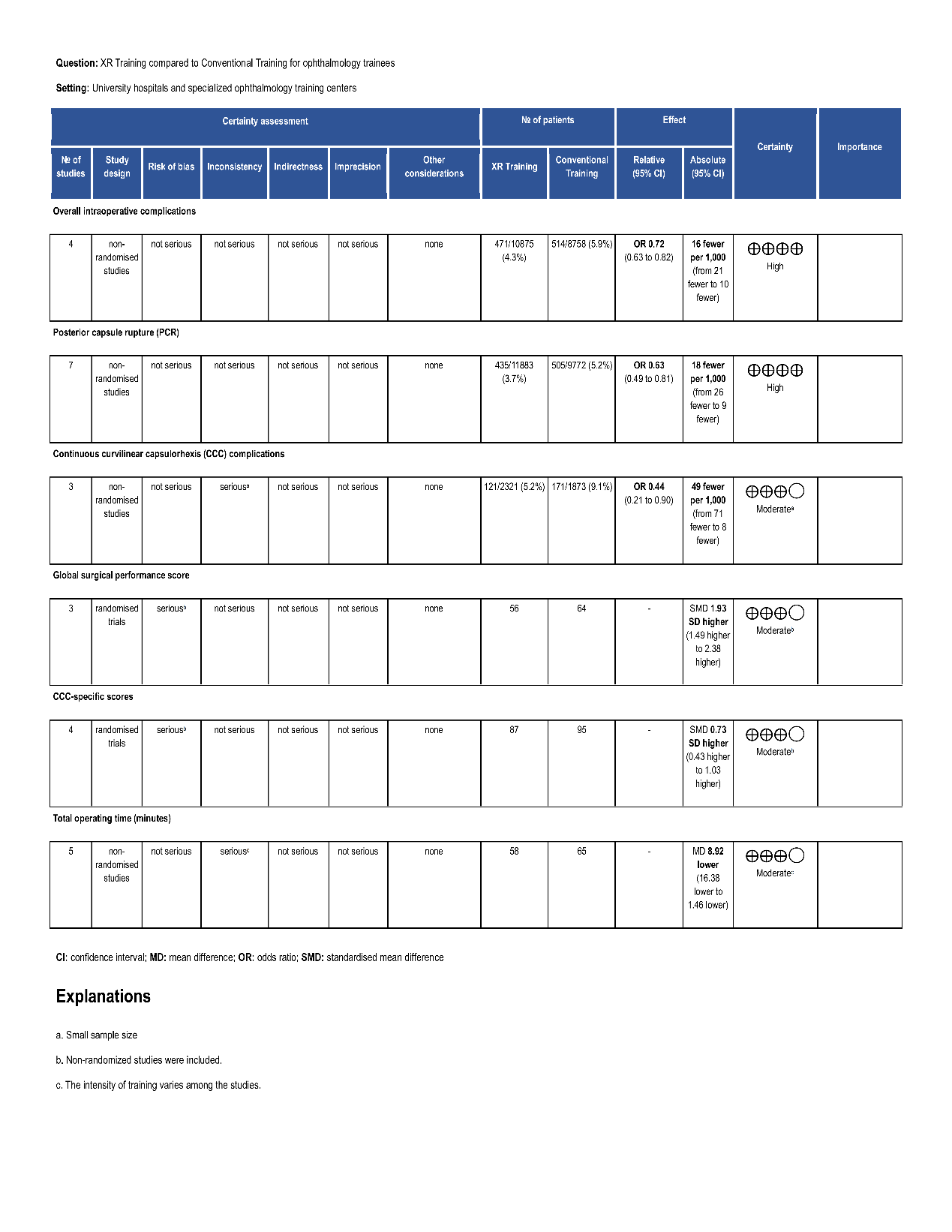


**Supplementary Figure 3. Summary of GRADE evidence quality assessment.**


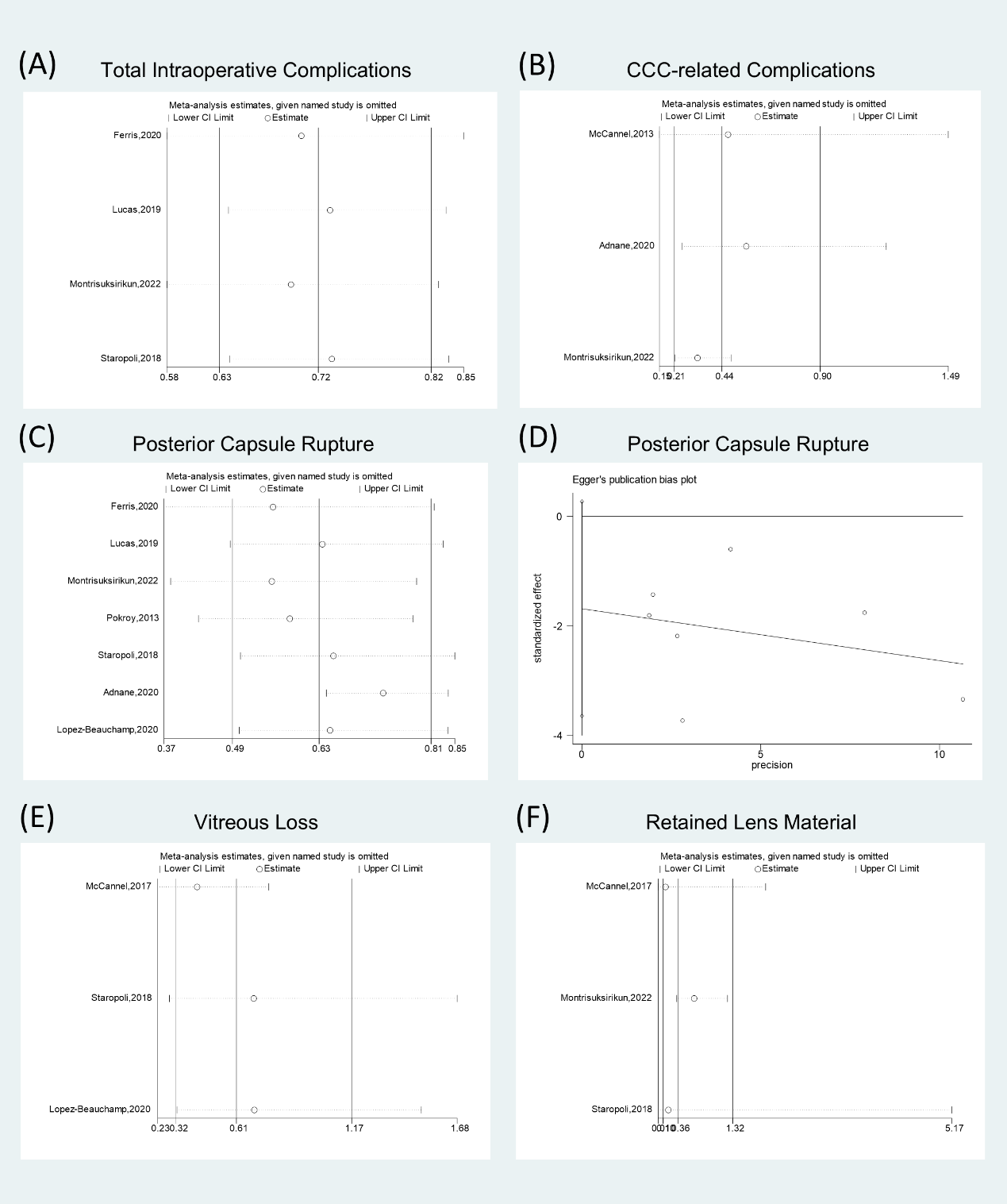


**Supplementary Figure 4. Sensitivity analyses and publication bias assessment for intraoperative complications.**

**(A)** Sensitivity analysis of total intraoperative complications. **(B)** Sensitivity analysis of continuous curvilinear capsulorhexis (CCC)-related complications. **(C)** Sensitivity analysis of posterior capsule rupture (PCR). **(D)** Publication bias assessment for PCR. **(E)** Sensitivity analysis of vitreous loss. **(F)** Sensitivity analysis of retained lens material. CCC: continuous curvilinear capsulorhexis; PCR: posterior capsule rupture.


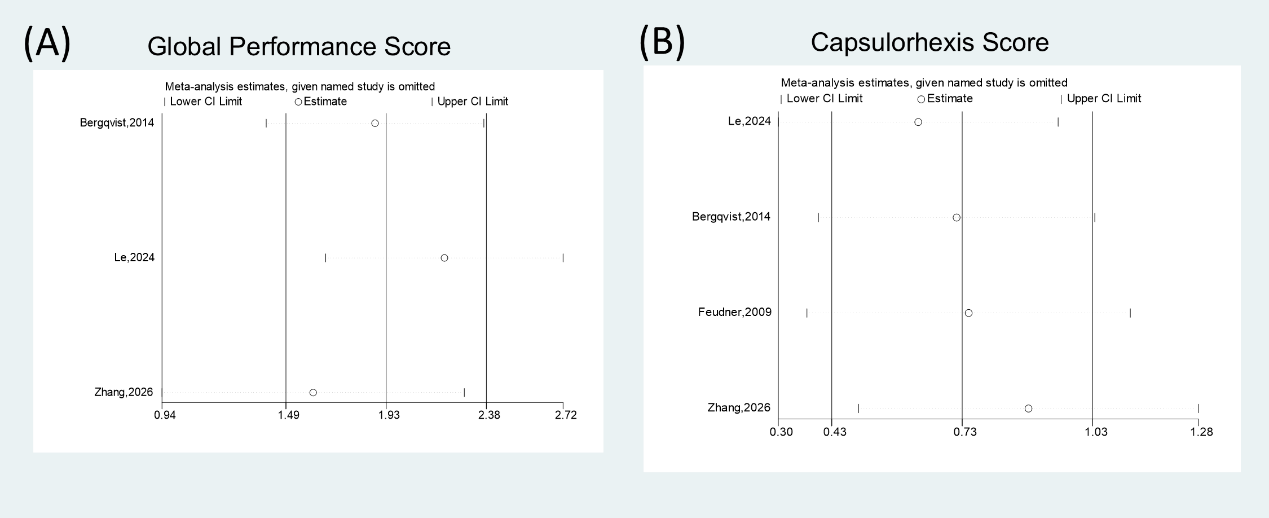


**Supplementary Figure 5. Sensitivity analyses for surgical performance scores.**

**(A)** Sensitivity analysis of Global Performance Score. **(B)** Sensitivity analysis of capsulorhexis score.


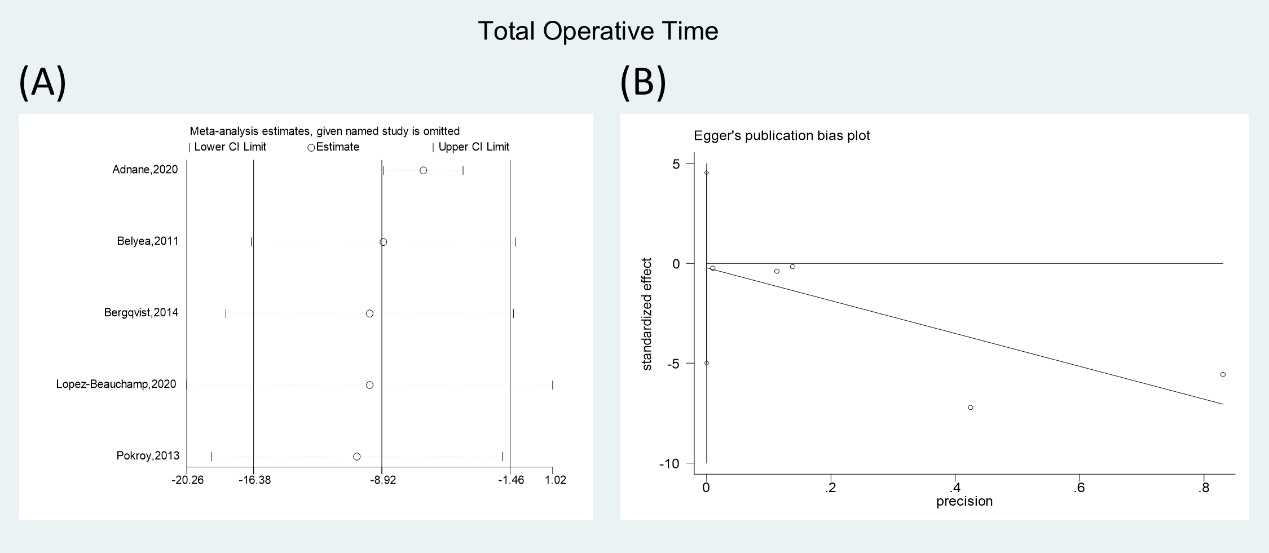


**Supplementary Figure 6. Sensitivity analysis and publication bias assessment for total operative time.**

**(A)** Sensitivity analysis of total operative time. **(B)** Publication bias assessment for total operative time.
